# Weekend admissions may be associated with poorer recording of long-term comorbidities: a prospective study of emergency admissions using administrative data

**DOI:** 10.1186/s12913-018-3668-7

**Published:** 2018-11-16

**Authors:** Therese Lloyd, Sarah R. Deeny, Adam Steventon

**Affiliations:** The Health Foundation, 90 Long Acre, London, WC2E 9RA UK

**Keywords:** Weekend effect, Mortality, Recording, Coding, Risk adjustment, Comorbidity, Administrative data

## Abstract

**Background:**

Many studies have investigated the presence of a ‘weekend effect’ in mortality following hospital admission, and these frequently use diagnostic codes from administrative data for information on comorbidities for risk adjustment. However, it is possible that coding practice differs between week and weekend. We assess patients with a confirmed history of certain long-term health conditions and investigate how well these are recorded in subsequent week and weekend admissions.

**Methods:**

We selected six long-term conditions that are commonly assessed when risk-adjusting mortality rates, via the Charlson and Elixhauser indices. Using Hospital Episode Statistics data from England for the period April 2009 to March 2011, we identified patients with the condition recorded at least twice, on separate emergency admissions. Then we assessed how often each condition was recorded on subsequent emergency admissions between April 2011 and March 2013. We then compared coding between week and weekend admissions using the Cochran-Mantel-Haenszel test, stratifying by hospital.

**Results:**

We studied 111,457 patients with chronic pulmonary disease, 106,432 with diabetes, 36,447 with congestive heart failure, 30,996 with dementia, 7808 with hemiplegia or paraplegia and 5877 with metastatic cancer. Across the entire week, between April 2011 and March 2013, coding completeness ranged from 89% for diabetes to 43% for hemiplegia/paraplegia. Compared with weekday admissions, congestive heart failure was less likely to be recorded as a secondary diagnosis at the weekend (odds ratio 0.92, 95% CI, 0.88 to 0.97), with smaller but statistically significant differences also detected for chronic pulmonary disease (odds ratio 0.96, 95% CI, 0.93 to 0.99) and diabetes (odds ratio 0.95, 95% CI 0.91 to 0.99). There was no statistically significant difference in recording between week and weekend admissions for dementia (odds ratio 1.04, 95% CI 0.97 to 1.11), hemiplegia/paraplegia (odds ratio 0.99, 95% CI 0.89 to 1.10) or metastatic cancer (odds ratio 1.04, 95% CI 0.90 to 1.20).

**Conclusions:**

Long-term conditions are often not recorded on administrative data and the lack of recording may be worse for weekend admissions. Studies of the weekend effect that rely on administrative data might have underestimated the health burden of patients, particularly if admitted at the weekend.

## Background

To date over 100 studies across a wide range of conditions and settings [1] have investigated the ‘weekend effect’, as first identified by Bell and Redelemeir in 2001. [2] While many studies have shown that mortality rates are higher amongst patients admitted to hospital during the weekend than those admitted during the week, little is still understood regarding its causes [1, 3]. There are two possibilities: either patients admitted at the weekend receive poorer quality care, or they have greater levels of health need that are not fully accounted for by the risk adjustment methods used. The need to untangle these issues has emerged as a particular priority in England, as policy makers and politicians seek to provide more consistent health services across the week [4–6].

Problems have arisen in part because studies have typically relied on administrative data, [2, 6–9] which were originally collected for the day-to-day management of health care (for example, for the reimbursement of hospital services), rather than research. While these data contain some important predictors of mortality (such as age and clinical diagnoses), they have limitations, [10, 11] including a lack of clinical detail on the severity of health conditions [12–14]. As severity might differ between patients admitted at the weekend and those admitted during the week, this difference may confound estimates of the weekend effect [7, 8, 15, 16]. Several recent studies have tried to address this limitation of administrative data by linking in additional information related to the severity of health conditions, such as the route of arrival to hospital [16]. These studies have often found a less pronounced weekend effect than those that applied conventional risk adjustment methods to administrative data alone [8, 16, 17]. Other studies have exploited clinical data that are specific to certain conditions, thus allowing adjustment for disease-specific measures of severity [18–20]. Those studies have lower generalisability than those based on administrative data, but intriguingly show a diminished weekend effect.

Common to studies that use administrative data to understand the weekend effect is the reliance on the same administrative data when assessing a patient’s comorbidities, [2, 6–9] for example using the Elixhauser list of comorbidities [21] or the Charlson list or weighted index score [22–24]. Both measures are considered predictive of death and have been validated numerous times [23–25].

The Charlson and Elixhauser measures are based on diagnoses recorded on the inpatient record yet those clinical diagnoses may be inaccurate, either because the source material for administrative data (for example, medical notes) are inaccurate or incomplete, or because errors occur when transcribing information from medical notes into the set of codes required by administrative data systems [26–29]. Studies that have evaluated coding accuracy across the entire week have found varying levels of accuracy, which could reflect variation in practice across health care systems or differences between coders used in particular studies [30]. A systematic review estimated the median diagnostic accuracy in coding (across all diagnosis fields) at 80.3% [30]. There is evidence that coding accuracy in administrative data has increased over time [30–33]. However, there remains evidence of differences in levels of recording of diagnoses in administrative datasets between NHS hospital trusts, [27] and within registry data [34]. Against this backdrop, it is surprising that such little attention has been paid to the possibility that coding accuracy might vary between weekend and weekday admissions, potentially biasing estimates of the weekend effect. To our knowledge, only one study has investigated that issue, [14] which was limited to stroke patients and, unlike most of the weekend effect studies, focussed on elective admissions rather than the emergency care pathway. Although the study produced valuable insights for stroke, it examined the accuracy of primary diagnosis recording, when typically studies of the weekend effect use information on secondary diagnoses.

The need for more detailed investigation is apparent from an apparently paradoxical observation: certain studies have found fewer [2, 8, 35] or similar levels of comorbidities [17] recorded for patients admitted at the weekend than for patients admitted during the week, even though some of these studies’ findings suggested that patients admitted at the weekend were sicker than those admitted during the week [8, 17]. To shed light on the issue, in this paper, we studied patients with a confirmed history of certain long-term health conditions, and tested empirically whether the likelihood of these conditions being recorded in administrative data differed depending on whether the admission occurred at the weekend or during the week. Our focus was on the conditions that feature in the Charlson and Elixhauser indices, since those commonly feature in the risk adjustment methods used to estimate the weekend effect from administrative data [2, 6–9, 13, 16]. By virtue of being included in these indices, the conditions are predictive of mortality following hospital admission, often being prevalent amongst the admitted population and having high associated mortality rates [21, 22]. We limited our study to long-term conditions that are very unlikely to be resolved between hospital visits. Therefore once recorded in the hospital data for a patient, these conditions should remain present and be recorded at subsequent hospital admissions.

## Methods

### Data set

We had access to data on inpatient admissions in England between 1 April 2009 and 31 March 2013 from Hospital Episode Statistics (HES), a national database containing administrative data from all NHS hospitals in England. Diagnoses are coded in the database using International Classification of Diseases, 10th Revision (ICD-10) codes. Within the data set, admissions are defined as a patient’s period of care within one hospital. If during the hospital stay a patient is under the care of multiple consultants, the admission will consist of several ‘finished consultant episodes’. Current guidance on clinical coding in England states that all clinically relevant conditions must always be coded for any inpatient episode in the administrative data [36]. Within HES inpatient records there are 20 diagnosis fields, the first of which documents the primary diagnosis, i.e. the main reason for the admission (primary diagnosis field), while the subsequent fields are used for recording comorbidities and complications (secondary diagnosis fields) [37]. As there is no ‘present on admission’ flag in the HES database, it is not possible to differentiate between conditions present on admission and complications that develop during the hospital stay.

### Inclusion and exclusion criteria

Consistent with other studies of the weekend effect, we restricted our study to emergency admissions, [13, 17, 35, 38–40] which are those that occur through the emergency department or via direct, urgent referrals from a general practitioner or other health professional. We further limited our analysis to admissions at acute non-specialist hospital trusts (a trust can comprise several hospitals), again consistent with many other studies in this area [8, 16, 35, 38]. We excluded emergency admissions that were transfers from another hospital, as in those cases the quality of coding might reflect the practices of several hospitals.

We included patients aged 18 or over, since the Charlson and Elixhauser indices were designed for use in adults [21–23]. We imposed a maximum age of 110, as some trusts code missing dates of birth as 1 January 1900 or 1 January 1901. Finally, we excluded private patients in NHS hospitals, and patients with sex or age missing in Hospital Episode Statistics. This meant that patients with human immunodeficiency virus were removed from our data set, since in those cases Hospital Episode Statistics do not contain date of birth [41].

### Creation of the study cohorts

The Charlson index contains 17 health conditions, while the Elixhauser index contains 32 [23]. From the combined set of Charlson and Elixhauser conditions, we selected those that are unlikely to be resolved within the 4 year time frame of our study, based on a discussion with a clinician. This produced six long-term conditions, namely chronic pulmonary disease, congestive heart failure, diabetes, metastatic cancer, dementia and hemiplegia/paraplegia. Of the six conditions, all but one are considered to always be clinically relevant to a patient’s care and are therefore subject to mandatory reporting within all inpatient episodes [36]. The exception is metastatic cancer, though this condition will often be clinically relevant to the patient’s admission, and so should also be recorded.

All these conditions are amalgamations of several diagnoses; for example, ‘chronic pulmonary disease’ includes both chronic obstructive pulmonary disease and asthma. Although both the Charlson and Elizhauser indices distinguish between complicated and uncomplicated diabetes, we assessed diabetes regardless of complications, to limit the potential for miscoding. We used the ICD-10 codes as defined by Quan et al. [23] and listed in Table 1.

**Table 1:** Conditions included in the study

Condition	Comorbidity list	International Classification of Diseases (ICD) 10th revision codes
Chronic pulmonary disease	Charlson and Elixhauser	I27.8, I27.9, J40.x-J47.x, J60.x-J67.x, J68.4, J70.1, J70.3
Congestive heart failure	Charlson and Elixhauser	I09.9, I11.0, I13.0, I13.2, I25.5, I42.0, I42.5-I42.9, I43.x, I50.x, P29.0
Diabetes (with or without complications)	Charlson and Elixhauser	E10.0, E10.1, E10.6, E10.8, E10.9, E11.0, E11.1, E11.6, E11.8, E11.9, E12.0, E12.1, E12.6, E12.8, E12.9, E13.0, E13.1, E13.6, E13.8, E13.9, E14.0, E14.1, E14.6, E14.8, E14.9, E 10.2-E10.8, E11.2--E 11.8, E12.2- E12.8, E13.2-E13.8, E14.2-E14.8
Metastatic cancer	Charlson and Elixhauser	C77.x-C80.x
Hemiplegia/paraplegia	Charlson and Elixhauser	G04.1, G11.4, G80.1, G80.2, G81.x, G82.x, G83.0-G83.4, G83.9
Dementia	Charlson	FOO.x-F03.x, F05.1, G30.x, G31.1

We examined each condition separately, producing six disease cohorts. In each case, we identified patients with the relevant condition recorded at least twice, on separate emergency admissions, between 1 April 2009 and 31 March 2011 (the pre-period). To identify patients with a confirmed history of the disease, the condition could have been recorded within any consultant episode, and within either the primary or secondary diagnosis fields. We then restricted our attention further to those patients who also experienced an emergency admission in the subsequent 2 years (between 1 April 2011 and 31 March 2013, the follow-up period). If a patient had several of the chronic conditions, they were included in several disease cohorts.

We examined how often each condition was recorded on inpatient emergency admissions data in the follow-up period in each of the disease cohorts. In doing so, we excluded admissions for which the relevant condition was recorded as the primary diagnosis since, in studies of the weekend effect, risk-adjustment methods commonly focus on secondary diagnoses (Table 2). We reasoned that, where the condition was not given as the primary diagnosis, it should be recorded in one of the secondary diagnosis fields. For admissions that contained multiple consultant episodes, we retained only the first episode, consistent with the risk adjustment method most appropriate when using the Charlson list or index on administrative datasets when there is no ‘present on admission’ flag (Table 2). Where a patient had multiple qualifying emergency admissions during the follow-up period, we selected the first.

**Table 2:** Background on the use of the Charlson and Elixhauser measures using HES data

Although both the Charlson and Elixhauser measures should ideally identify comorbidities by including information reported at prior admissions, [24, 40, 53] often only data from the ‘index’ hospital admission (i.e. the admission of interest) is used to determine comorbidities at the time of admission [54, 55]. When risk adjusting using the Charlson list or index, data can be limited to diagnoses recorded in the first episode of the index hospital admission, to avoid the risk of including complications that arose during the hospital admission. The Elixhauser measure, however, was designed for use on databases that cannot distinguish between comorbidities and complications and limits its set of conditions to either chronic conditions or acute conditions that are unlikely to be potential complications [21]. When risk adjusting using the Elixhauser list, comorbidity data from all episodes within a hospital stay can be used. Depending on the purpose of the risk adjustment, one either includes only secondary diagnoses from the index admission [16, 58] (on the assumption that these fields contain concurrent conditions that may impact on mortality rates, over and above the primary reason for the admission) or all diagnosis fields.	

### Statistical analysis

We defined the weekend as running from midnight Saturday morning to midnight Monday morning, and weekday as all other times. We then compared how often the relevant condition was recorded during week and weekend admissions, by calculating Mantel-Haenszel common odds ratios [42]. The statistical significance of the common odds ratios was assessed using the Cochran-Mantel-Haenszel test [43, 44]. This allowed a non-parametric approach that does not require any modelling assumptions. The analysis was stratified at the level of the hospital trust, since coding practices might systematically differ between trusts. This in effect creates individual odds ratios for each hospital trust, then computes a common odds ratio across all hospital trusts by weighting the odds ratios across the trusts. We used PROC FREQ in SAS Enterprise Guide 7.1 and report the Mantel-Haenszel common odds ratio estimators, as these are less affected by sparse data and in particular zero cells and are often more stable than the alternative Woolf’s method (logit estimation in SAS) [45].

The Cochran-Mantel-Haenszel test assumes that the odds ratios for each hospital trust are similar. If the odds ratios vary between trusts, the test has low statistical power (in other words, it is unlikely to detect differences in recording practices between week and weekend admissions, even if they exist) [46]. Therefore, the Cochran-Mantel-Haenszel test is often supplemented with the Breslow-Day test, which examines whether the odds ratios are homogeneous across trusts [45]. If the Breslow-Day test is significant, then the odds ratios vary between trusts, and the Cochran-Mantel-Haenszel test will have low statistical power; in that instance, an insignificant result from Cochran-Mantel-Haenszel test is unlikely to be informative.

### Sensitivity analyses

As there are different methods of identifying the comorbidities when risk adjusting, we performed sensitivity analyses that reflected these alternative methods.

Firstly, we examined emergency admissions in the follow-up period and compared week and weekend admissions in terms of whether the relevant condition was recorded in any diagnosis field (primary or secondary) in the first consultant episode. In this analysis, we included admissions, regardless of whether the relevant condition was recorded in the primary diagnosis field.

Secondly, we examined whether there were differences in recording between weekend and weekday emergency admissions within the follow-up period, when we included all diagnosis fields from all consultant episodes during a patient hospital stay.

We also examined whether differences between weekend and weekday recording existed specifically for admissions that occurred near the end of life. It could be that hospital teams spend less time trying to identify and record co-morbidities for patients who have died. Since patients attending at the weekend are more likely to die, this could bias any analysis of the ‘weekend effect’. Consistent with many studies of the weekend effect, we examined admissions that occurred within 30 days of death, and restricted the analysis to deaths that occurred in hospital [8, 16, 17, 47]. We excluded admissions where the relevant condition was given as the primary diagnosis, and compared week and weekend admissions in terms of whether the relevant condition was recorded in the secondary diagnosis fields within the first episode. Where a patient had several admissions in the 30 days prior to dying, the first of these admissions was selected.

## Results

**Fig. 1:**
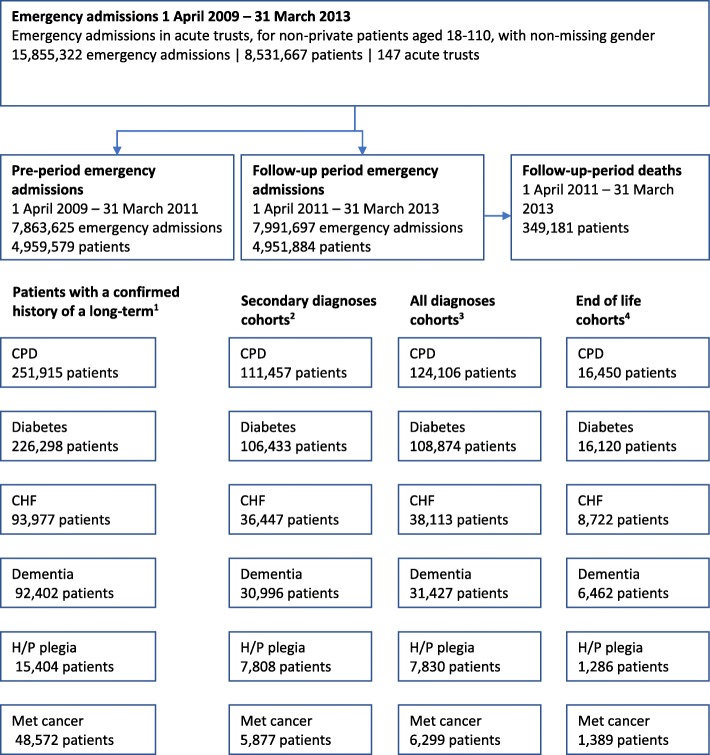
Flow chart. ^1^Patients with a confirmed history of a long-term condition: Patients with at least 2 separate emergency admissions in the pre-period at which the comorbidity was recorded. ^2^Secondary diagnoses cohorts: Patients with at least two admissions in the pre-period where the comorbidity was recorded and a post-period admission where the diagnosis was not recorded as the primary diagnosis. ^3^All diagnoses cohorts: Patients with at least 2 admissions in the pre-period where the comorbidity was recorded and a post-period admission. ^4^End of life cohorts: Patients with at least two admissions in the pre-period where the comorbidity was recorded and a post-period admission within 30 days of death where the diagnosis was not recorded as the primary diagnosis. CPD=chronic pulmonary disease; CHF=congestive heart failure; H/P plegia=hemiplegia / paraplegia; Met cancer=metastatic cancer

Between 1 April 2009 and 31 March 2011, there were 7,863,625 inpatient emergency admissions in England for 4,959,579 adult patients at 147 acute non-specialist trusts that satisfied our inclusion and exclusion criteria (Fig. 1).

The number of patients identified as having a confirmed history of a long-term condition within the pre-period were as follows: 251,915 patients with chronic pulmonary disease, 226,298 with diabetes, 93,977 with congestive heart failure, 92,404 with dementia, 15,404 with hemiplegia or paraplegia and 48,572 with metastatic cancer (Fig. 1).

After restricting our attention to patients with a confirmed history of the relevant condition and an emergency admission during the follow-up period (for which the relevant condition was not listed as the primary diagnosis), there were 111,457 patients with chronic pulmonary disease, 106,433 with diabetes, 36,447 with congestive heart failure, 30,996 with dementia, 7808 with hemiplegia or paraplegia and 5877 with metastatic cancer (Fig. 1 and Table 3).

**Table 3:** Number of patients in each disease cohort

Condition	Number of patients^a^		
	Admitted on weekday	Admitted at weekend	Total
Chronic pulmonary disease	83,938	27,519	111,457
Diabetes	80,419	26,014	106,433
Congestive heart failure	27,244	9203	36,447
Dementia	22,115	8881	30,996
Hemiplegia / paraplegia	5755	2053	7808
Metastatic cancer	4503	1374	5877

^a^Patients with at least two emergency admissions with the relevant condition recorded between 2009 and 2011 and a further admission in 2011–2013 where the relevant condition was not the primary diagnosis

The patients in these disease cohorts had a median age ranging from 69 years for metastatic cancer to 85 years for dementia (Table 4). The cohorts had similar proportions of men and women, except for dementia, chronic pulmonary disease and hemiplegia/paraplegia, for which 36, 43 and 55% of patients were men, respectively. The 30-day mortality rates ranged from 6.0% for patients with diabetes to 13.3% for patients with metastatic cancer.

**Table 4:** Descriptive statistics for each disease cohort

Condition	Number of patients^a^	Male (% of patients)	Age in years: median (interquartile range)	Died within 30 days (% of patients)
Chronic pulmonary disease	111,457	43.2	73 (57–82)	6.5
Diabetes	106,433	50.5	75 (63–82)	6.0
Congestive heart failure	36,447	49.2	81 (73–87)	10.1
Dementia	30,996	35.5	85 (80–90)	10.9
Hemiplegia/paraplegia	7808	54.7	72 (59–81)	6.4
Metastatic cancer	5877	49.3	69 (60–77)	13.3

^a^Patients with at least two emergency admissions with the relevant condition recorded between 2009 and 2011 and a further admission in 2011–2013 where the relevant condition was not the primary diagnosis

Levels of recording varied across the conditions. After excluding admissions for which diabetes was listed as the primary diagnosis, and looking across both week and weekend admissions, 89% of admissions for the diabetes cohort had diabetes recorded on a secondary diagnosis field. Hemiplegia/paraplegia had the poorest recording, with only 43% of relevant admissions containing this diagnosis, followed by congestive heart failure at 53% of relevant admissions (Table 5).

**Table 5:** Comparison of secondary diagnoses between weekend and weekday emergency admissions (secondary diagnosis fields within the first episode)

Condition	Percentage of admissions with the diagnosis recorded in the secondary diagnosis fields^a^			Common odds ratio^b^ for weekend versus week (95% confidence interval)	Cochran-Mantel-Haenszel test *p*-value^c^	Breslow-Day test *p*-value^d^
	Weekday admissions	Weekend admissions	Total			
Chronic pulmonary disease	76.44	75.82	76.29	0.962 (0.932 to 0.994)	0.020	0.094
Diabetes	89.20	88.72	89.08	0.950 (0.909 to 0.994)	0.026	0.320
Congestive heart failure	53.27	51.32	52.78	0.922 (0.878 to 0.967)	0.001	0.565
Dementia	83.95	84.44	84.09	1.036 (0.967 to 1.109)	0.313	0.717
Hemiplegia / paraplegia	43.09	43.25	43.14	0.990 (0.892 to 1.099)	0.852	0.031
Metastatic cancer	75.64	76.35	75.80	1.042 (0.902 to 1.204)	0.579	0.473

^a^Patients with at least two emergency admissions with the relevant condition recorded between 2009 and 2011 and a further admission in 2011–2013 where the relevant condition was not the primary diagnosis

^b^Common odds ratio computed from trust-level odds ratios

^c^Cochran-Mantel-Haenszel test for odds ratio not equal to one

^d^Breslow-Day test for homogeneity of odds ratios across trusts

Compared with weekday admissions, chronic pulmonary disease was less likely to be recorded as a secondary diagnosis on weekend admissions (odds ratio 0.96, 95% confidence interval (CI), 0.93 to 0.99), and similar patterns were found for diabetes (odds ratio 0.95, 95% CI 0.91 to 0.99) and congestive heart failure (odds ratio 0.92, 95% CI, 0.88 to 0.97). There was no statistically significant difference between weekends and weekdays for dementia, hemiplegia/paraplegia or metastatic cancer. With the exception of hemiplegia/paraplegia, the Breslow-Day test found no evidence that odds ratios were heterogenous across trusts.

### Sensitivity analyses

When we included admissions with the condition recorded as the primary diagnosis, and examined coding across primary and secondary diagnosis fields within the first episode, we found similar results to the main analyses. Chronic pulmonary disease was less likely to be recorded on weekend than weekday admissions (odds ratio 0.96, 95% CI, 0.93 to 1.00), as were diabetes (odds ratio 0.95, 95% CI, 0.90 to 0.99) and congestive heart failure (odds ratio 0.91, 95% CI, 0.87 to 0.95) – see Tables 6 and 7. As in the main analysis, dementia, hemiplegia/paraplegia and metastatic cancer did not show a statistically significant difference between weekends and weekdays; and only hemiplegia/paraplegia showed evidence of heterogeneity in odds ratios across hospital trusts.

**Table 6:** Number of patients in each disease cohort: regardless of primary diagnosis

Condition	Number of patients^a^		
	Admitted on weekday	Admitted at weekend	Total
Chronic pulmonary disease	93,482	30,624	124,106
Diabetes	82,325	26,549	108,874
Congestive heart failure	28,551	9562	38,113
Dementia	22,430	8997	31,427
Hemiplegia / paraplegia	5776	2054	7830
Metastatic cancer	4853	1446	6299

^a^Patients with at least two emergency admissions with the relevant condition recorded between 2009 and 2011 and a further admission in 2011–2013

**Table 7:** Comparison of diagnoses between weekend and weekday emergency admissions: regardless of primary diagnosis (all diagnosis fields within the first episode)

Condition	Percentage of admissions with the diagnosis recorded in any field^a^			Common odds ratio^b^ for weekend versus week (95% confidence interval)	Cochran-Mantel-Haenszel test *p*-value^c^	Breslow-Day test *p*-value^d^
	Weekday admissions	Weekend admissions	Total			
Chronic pulmonary disease	79.69	79.16	79.56	0.964 (0.934 to 0.996)	0.027	0.051
Diabetes	89.61	89.09	89.48	0.946 (0.904 to 0.989)	0.015	0.312
Congestive heart failure	56.79	54.46	56.20	0.906 (0.865 to 0.950)	0.000	0.563
Dementia	84.27	84.75	84.41	1.036 (0.967 to 1.109)	0.312	0.647
Hemiplegia / paraplegia	43.49	43.62	43.52	0.989 (0.891 to 1.097)	0.835	0.019
Metastatic cancer	77.75	77.80	77.76	1.004 (0.869 to 1.160)	0.960	0.543

^a^Patients with at least two emergency admissions with the relevant condition recorded between 2009 and 2011 and a further admission in 2011–2013

^b^Common odds ratio computed from trust-level odds ratios

^c^Cochran-Mantel-Haenszel test for odds ratio not equal to one

^d^Breslow-Day test for homogeneity of odds ratios across trusts

Out of the patients listed in Table 6, between 43 and 53% of patients (depending on disease cohort) were under the care of more than one consultant during their emergency hospital stay and therefore had more than one finished consultant episode. This is higher than for admissions in general, where the vast majority of admissions contain only one episode [48, 49]. This is to be expected, given that our cohorts are limited to emergency admissions and to patients who are likely to have more complex needs due to their long-term conditions.

When we included all diagnoses recorded over a patient’s hospital stay, i.e. both primary and secondary diagnoses, over all episodes, the results were similar to those of the main analysis. Chronic pulmonary disease was less likely to be recorded on weekend than weekday admissions (odds ratio 0.96, 95% CI, 0.93 to 0.99), as were diabetes (odds ratio 0.94, 95% CI, 0.90 to 0.98) and congestive heart failure (odds ratio 0.90, 95% CI, 0.86 to 0.95), see Table 8. Dementia, hemiplegia/paraplegia and metastatic cancer did not show a statistically significant difference between weekends and weekdays. Chronic pulmonary disease and hemiplegia/paraplegia showed evidence of heterogeneity in odds ratios across hospital trusts.

**Table 8:** Comparison of diagnoses between weekend and weekday emergency admissions: regardless of primary diagnosis (all diagnosis fields from all episodes)

Condition	Percentage of admissions with the diagnosis recorded in any field^a^			Common odds ratio^b^ for weekend versus week (95% confidence interval)	Cochran-Mantel-Haenszel test *p*-value^c^	Breslow-Day test *p*-value^d^
	Weekday admissions	Weekend admissions	Total			
Chronic pulmonary disease	80.40	79.77	80.25	0.958 (0.927 to 0.989)	0.009	0.028
Diabetes	90.28	89.72	90.14	0.940 (0.898 to 0.985)	0.009	0.316
Congestive heart failure	58.75	56.34	58.15	0.902 (0.861 to 0.946)	0.000	0.558
Dementia	85.25	85.47	85.31	1.018 (0.949 to 1.092)	0.616	0.582
Hemiplegia / paraplegia	44.13	44.30	44.18	0.991 (0.893 to 1.099)	0.858	0.034
Metastatic cancer	78.63	78.56	78.62	1.000 (0.863 to 1.157)	0.995	0.620

^a^Patients with at least two emergency admissions with the relevant condition recorded between 2009 and 2011 and a further admission in 2011–2013

^b^Common odds ratio computed from trust-level odds ratios

^c^Cochran-Mantel-Haenszel test for odds ratio not equal to one

^d^Breslow-Day test for homogeneity of odds ratios across trusts

When we restricted our attention to emergency admissions within 30 days of death (Table 9), the proportion of comorbidities that were recorded was in general higher than in the main analysis cohorts (Tables 5 and 10). We again found that diabetes was less likely to be recorded on weekend than weekday admissions (odds ratio 0.89, 95% CI, 0.79 to 0.99) and likewise for congestive heart failure (odds ratio 0.89, 95% CI, 0.80 to 0.98). Chronic pulmonary disease showed an odds ratio of 0.96 as in the main analysis, but results were not statistically significant (95% CI, 0.88 to 1.05) (Tables 9 and 10). Dementia, hemiplegia/paraplegia and metastatic cancer did not show a statistically significant difference between weekends and weekdays. For the dementia cohort, the Breslow-Day test indicated that the odds ratios are heterogeneous across trusts.

**Table 9:** Number of patients in each disease cohort: admissions within 30 days of death

Condition	Number of patients^a^		
	Admitted on weekday	Admitted at weekend	Total
Chronic pulmonary disease	12,041	4409	16,450
Diabetes	11,847	4273	16,120
Congestive heart failure	6420	2302	8722
Dementia	4542	1920	6462
Hemiplegia / paraplegia	925	361	1286
Metastatic cancer	1029	360	1389

^a^Patients with at least two emergency admissions with the relevant condition recorded between 2009 and 2011 and a further admissions within 30 days of death in 2011–2013 where the relevant condition was not the primary diagnosis

**Table 10:** Comparison of secondary diagnoses between weekend and weekday emergency admissions within 30 days of death (secondary diagnosis fields within the first episode)

Condition	Percentage of admissions with the diagnosis recorded in the secondary diagnosis fields^a^			Common odds ratio^b^ for weekend versus week (95% confidence interval)	Cochran-Mantel-Haenszel test *p*-value^c^	Breslow-Day test *p*-value^d^
	Weekday admissions	Weekend admissions	Total			
Chronic pulmonary disease	82.44	81.95	82.31	0.960 (0.876 to 1.051)	0.371	0.189
Diabetes	89.34	88.18	89.03	0.885 (0.792 to 0.989)	0.030	0.230
Congestive heart failure	62.57	59.99	61.89	0.887 (0.803 to 0.980)	0.019	0.481
Dementia	87.87	88.85	88.16	1.086 (0.916 to 1.287)	0.343	0.025
Hemiplegia / paraplegia	45.19	44.32	44.95	0.944 (0.725 to 1.230)	0.672	0.281
Metastatic cancer	86.01	84.72	85.67	0.907 (0.630 to 1.306)	0.599	0.112

^a^Patients with at least two emergency admissions with the relevant condition recorded between 2009 and 2011 and a further admission within 30 days of death in 2011–2013 where the relevant condition was not the primary diagnosis

^b^Common odds ratio computed from trust-level odds ratios

^c^Cochran-Mantel-Haenszel test for odds ratio not equal to one

^d^Breslow-Day test for homogeneity of odds ratios across trusts

## Discussion

Our study shows that common long-term conditions that are always considered clinically relevant to the care received in hospital are often poorly recorded in administrative data, even when they are subject to mandatory coding [36]. While diabetes was recorded in 89% of emergency admissions for patients with a confirmed history of diabetes, for patients with hemiplegia/paraplegia and congestive heart failure, these conditions were only recorded on 43 and 53% of admissions, respectively (across both weekend and weekday admissions). These findings will have implications for all studies that apply risk adjustment to administrative data.

Coding was less complete for some long-term conditions at the weekend compared with during the week. Patients with chronic pulmonary disease were less likely to have their condition recorded on weekend versus weekday admissions (odds ratio 0.96, 95% CI 0.93–0.99), and similar patterns were found for diabetes (odds ratio 0.95, 95% CI 0.91–0.99) and congestive heart failure (odds ratio 0.92, 95% CI 0.88–0.97), though not for dementia, hemiplegia/paraplegia or metastatic cancer. These findings were consistent across hospital trusts and robust to sensitivity analysis, although chronic pulmonary disease no longer reached statistical significance in the cohort of patients who died within 30 days of an emergency admission.

The results of our sensitivity analyses show that the difference in recording between weekend and weekday detected in chronic pulmonary disease, diabetes and congestive heart failure was consistent across different methods of identifying comorbidities in the data. The results were similar, independently of whether we used only secondary diagnoses within the first episode of a patient’s admission, all diagnoses within the first episode, or all diagnoses within a hospital stay. Even though the examined long-term conditions should have been recorded on each episode of a patient’s stay, the proportion of patients with a recorded comorbidity did in general increase when including more diagnosis fields. Diabetes had the smallest variation (89.1% using only secondary diagnoses, 89.5% using all diagnoses in the first episode and 90.1% using all diagnoses within the patient stay, across the whole week), while the largest variation in recording was for congestive heart failure (52.8% using only secondary diagnoses, 56.2% using all diagnoses in the first episode and 58.2% using all diagnoses within the patient stay, across the whole week). However, the advantage of including more data fields needs to be weighed against the risk of including potential complications when using for example the Charlson list. The odds ratios are broadly similar across the methods, therefore there is no evidence that either method introduces more bias in the recording of comorbidities between weekend and weekday.

When comparing the end of life cohorts to the main analysis cohorts, the proportion of comorbidities that were recorded was in general higher than in the overall population of patients with a confirmed history of the disease, the exception being diabetes (89.0% vs 89.1% in the main analysis). There is therefore no evidence that comorbidities are in general less well recorded for patients admitted within the last month of their life. However, the point estimate odds ratio between weekend and weekdays for diabetes was lower in the end of life cohort than in the main analysis (odds ratio 0.88 vs 0.95). The confidence intervals were larger than in the main analysis due to much smaller sample sizes (Fig. 1), but was still statistically significant. The same pattern was seen for patients with a confirmed history of congestive heart failure (odds ratio 0.89 vs 0.92), indicating that the difference in recording weekend to weekday of some long-term conditions may be more pronounced for patients nearing their end of life. For chronic pulmonary disease, the point estimate odds ratio was similar to that in the main analysis, but due to wider confidence intervals, the difference was no longer statistically significant.

### Possible mechanisms

In some rare cases, there may be valid reasons why conditions are no longer present in subsequent admissions. For example, asthma may resolve over time. In some cases, the condition might be legitimately recorded in several ways. For example, hemiplegia/paraglegia may be recorded as previous stroke, paralytic stroke or brain injury, and these may not all be mapped to the ICD-10 codes used to define hemiglegia/paraplegia in the Elixhauser or Charlson indices. However, it is hard to see how these considerations could affect differences in recording between week and weekend admissions. Alternative explanations are more likely. For example, there are different staffing arrangements at the weekend compared with during the week; with fewer consultants, [38] and nurses [20] per emergency admission at the weekend, leading to more “cross-covering” by consultants (where consultants treat patients outside their main specialty). There may also be more temporary or agency staff on duty at the weekend. Such staffing arrangements might mean that diagnoses are less frequently recognised as clinically relevant during weekend admissions and subsequently not recorded. There may also be less awareness of the mechanisms by which the hospital trust is reimbursed for the care provided, potentially meaning that certain conditions associated with higher treatment payments are recorded less at the weekend [30]. Non-clinical services, such as a medical records department, may also be reduced in many hospitals at the weekend, leading to increased reliance on other information sources for recording of comorbidities at admission, such as patient reporting. This may be particularly difficult if the patient is older with many comorbidities, and might not be able to identify or remember all clinically relevant conditions. Any of these factors might have led to reduced data quality for weekend than week admissions. However, since there was little evidence of variability in the lower odds of recording at weekends across trusts, it is possible that there is a shared mechanism.

### Comparison with previous work

The only study that, to our knowledge, has investigated whether coding accuracy varies between week and weekend admissions was limited to stroke patients and focussed on primary diagnoses for elective admissions [14]. Those authors found some differences in coding between weekend and weekday admissions; strokes with low expected case fatality were more likely to be miscoded as acute stroke during the week than for similar admissions at the weekend, corroborating our findings that there were recording differences between the weekend and the week. Another study examined overall accuracy in recording of comorbidities over the whole week, but did not compare week and weekend admissions. This found that the accuracy of recording ranged from 36% for paraplegia to 76% for uncomplicated diabetes [27, 50] – lower than in our study, potentially because patients were identified as having these conditions on the basis of a diagnosis code being present on a single admission or because coding quality has improved since 2008–09. To our knowledge, this is the first study to examine variation in recording of comorbidities between weekend and weekday admissions using administrative data.

### Strengths and limitations

By selecting patients based on the diagnoses recorded across multiple admissions and then assessing prospectively whether these conditions were recorded in subsequent emergency admissions, we reduced the risk of erroneously identifying patients as having a condition, compared with other approaches. We selected long-term conditions that are commonly used in risk adjustment models, which we argue are non-reversible and therefore should continue to be recorded in hospital data. All but dementia feature in both the Charlson and Elixhauser indices, which underpin common approaches to risk adjustment. There are several available ‘translations’ for the ICD-10 codes involved, [23, 25, 27] and we used the list by Quan et al., [23] which encompasses a broader range of codes than other definitions.

As the Hospital Episode Statistics dataset is collected primarily for billing purposes our findings will also be relevant to similar administrative datasets collected for the same reason.

Our robust method for identifying patients with comorbidities limited our analyses to only six long-term conditions. We could not consider the coding of other common comorbidities, including acute conditions, which might contribute towards excess mortality at the weekend. Therefore, we were also not able to quantify the impact of differences in recording of comorbidities on estimates of the weekend effect.

We examined a national dataset of emergency hospital admissions for adults but our results apply only to patients with the conditions recorded on at least two separate admissions over a two-year period, who then experienced a subsequent admission. Our study population was therefore likely to be sicker and had a higher risk of 30-day mortality than most patients admitted to hospital, ranging from 6.0 to 13.3% across conditions, and higher than observed previously in the weekend effect literature [35].

As all but one of the studied long-term conditions are subject to mandatory reporting, [36] it was reasonable to assume that the conditions should be recorded regardless of a patient’s characteristics (e.g. age), and so we did not adjust for those characteristics in our statistical analysis. Instead, we adopted a non-parametric approach, which removed the need to make assumptions about the nature of the relationship between the variables. We stratified by hospital trust to allow for systematic differences in coding between hospital trusts, since those are indicated from the existing literature. However, we did not adjust for the month of admission, even though coding depth (i.e. the average number of diagnostic fields completed) has in general increased with time [27]. We reasoned that there are unlikely to be seasonal differences in the numbers of week and weekend admissions.

Our study examined the quality of recording during the period 2011 to 2013. Although there is no reason to think that the relative accuracy of weekend to weekday recording has improved since, we were not able to investigate this, nor did we examine whether recording changed over the course of the two-year period, as this was too short a period to do such an analysis justice.

Recent studies on variation in mortality have gone beyond dichotomous differences in weekend and weekday rates, instead examining differences across the days of the week and across time of the day [8, 18, 47, 51, 52]. However, this study was primarily based on the hypothesis that there may be differences in recording due to differences in staffing between the weekend and weekdays, and so the study design reflects this, thereby allowing for a larger sample size than if the study examined each day separately.

### Implications of our findings

These findings have implications for all studies that use administrative data for risk adjustment, including many observational studies, predictive models and resource allocation methods. In particular, our study shows that certain long-term conditions are less likely to be recorded at the weekend than during the week, adding further potential insight into the current controversy surrounding the weekend effect.

Researchers may have underestimated the burden of illness for weekend admissions compared with weekday admissions, thus exaggerating differences in risk-adjusted mortality rates. It was not possible for us to quantify the size of the impact, particularly because only a small sample of the conditions that are routinely included in risk adjustment could be assessed using our method. Although the differences in recording between weekend and weekdays were slight, we observed that they were larger for patients nearing the end of life.

Additional studies would be needed to determine whether there are differences between weekend and weekday recordings of other comorbidities used in standard risk adjustment and the extent to which these biases would impact on the ‘weekend effect’. For example, a study comparing recorded conditions in administrative hospital data with general practice medical records may bring further insight. Further studies could also examine the causes for the relatively poor recording, for example whether they result from deficiencies in the underlying medical notes or the way in which this information is transcribed into administrative data. There might be implications for the definition of the diagnosis codes included in risk-adjustment, if certain health conditions are not being consistently recorded within the existing sets of defined diagnosis codes.

In the meantime, the design of studies in this area could be improved to reflect the deficiencies we have identified within the administrative data. For example, studies could use a “lookback” period, thus combining diagnostic information from prior admissions to identify the presence of long-term health conditions [53–57]. Studies are often restricted to using diagnostic data from only the index admission, which may be more predictive of in-hospital mortality [57] but may also, as we show, result in systematic biases.

## Conclusion

Our findings add to the growing literature suggesting that caution is needed when using administrative data to estimate variation in patient outcomes. A reliance on recording of comorbidities in patient datasets may mean that commonly used risk adjustment methods such as Charlson or Elixhauser underestimate the illness of patients admitted, and that this may be particularly the case during the weekends, with potential implications for estimates of the weekend effect.

## Abbreviations

CI: Confidence interval
ICD: International Classification of Diseases
ICD-10: International Classification of Diseases, 10th Revision
